# Preserved 2-y Liver Transplant Outcomes Following Simultaneous Thoracoabdominal DCD Organ Procurement Despite Effects on Liver Utilization Rate

**DOI:** 10.1097/TXD.0000000000001528

**Published:** 2023-10-20

**Authors:** Steven A. Wisel, Justin A. Steggerda, Carrie Thiessen, Garrett R. Roll, Qiudong Chen, Jason Thomas, Bhupinder Kaur, Pedro Catarino, Joanna Chikwe, Irene K. Kim

**Affiliations:** 1 Department of Surgery, Comprehensive Transplant Center, Cedars-Sinai Medical Center, Los Angeles, CA.; 2 Division of Transplant Surgery, University of Wisconsin, Madison, WI.; 3 Division of Transplantation, University of California, San Francisco, San Francisco, CA.; 4 Department of Cardiac Surgery, Smidt Heart Institute, Cedars-Sinai Medical Center, Los Angeles, CA.

## Abstract

**Background.:**

Current techniques for donation after circulatory determination of death (DCD) heart procurement, through either direct procurement and machine perfusion or thoracoabdominal normothermic regional perfusion (NRP), have demonstrated excellent heart transplant outcomes. However, the impact of thoracoabdominal DCD (TA-DCD) heart procurement on liver allograft outcomes and utilization is poorly understood.

**Methods.:**

One hundred sixty simultaneous heart and liver DCD donors were identified using the United Network for Organ Sharing/Organ Procurement and Transplantation Network database between December 2019 and July 2021. Liver outcomes from TA-DCD donors were stratified by heart procurement technique and evaluated for organ utilization, graft survival, and patient survival. Results were compared with abdominal-only DCD (A-DCD; n = 1332) and donation after brain death (DBD; n = 12 891) liver transplants during the study interval. Kaplan-Meier methods with log-rank testing were used to evaluate patient and graft survival.

**Results.:**

One hundred thirty-three of 160 livers procured from TA-DCD donors proceeded to transplant. TA-DCD donors were younger (mean 28.26 y; *P* < 0.0001) with lower body mass index (mean 26.61; *P* < 0.0001) than A-DCD and DBD donors. TA-DCD livers had equivalent patient survival ( *P* = 0.893) and superior graft survival (*P* = 0.009) compared with A-DCD. TA-DCD livers had higher rates of organ discard for long warm ischemia time (37.0%) than A-DCD (20.5%) and DBD (0.5%; *P* < 0.0001), with direct procurement and machine perfusion procurements leading to a higher discard rate (18.5%) than NRP procurements (7.4%).

**Conclusions.:**

Liver transplants after TA-DCD donation demonstrated equivalent patient outcomes and excellent graft outcomes. NRP procurements resulted in the lowest rate of organ discard after DCD donation and may represent an optimal strategy to maximize organ utilization.

## Graphical abstract

**Figure:**
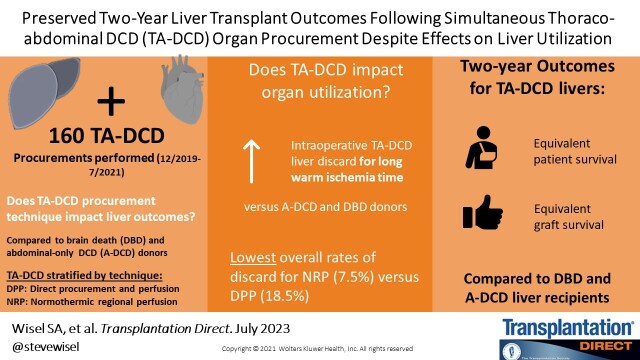

Although common practice in Europe and Australia, heart transplantation from donation after circulatory death (DCD) donors rapidly entered clinical practice in the United States in 2019 with the Donation After Cardiac Death Heart Trial (NC03831048).^1-4^ Cardiac DCD procurement is currently accomplished by either direct procurement and immediate machine perfusion or thoracoabdominal normothermic regional perfusion (NRP). Both techniques have shown excellent short-term heart transplant results, with prospective monitoring of cardiac outcomes as part of the Donation After Cardiac Death Heart Trial.^1,2^

The increasing frequency of cardiac DCD procurement, which significantly modifies existing DCD protocols, requires that abdominal transplant surgeons are aware of the logistics and technical considerations required for thoracoabdominal DCD (TA-DCD) procurements, either performed by direct procurement and perfusion (DPP) or NRP. The DPP strategy uses rapid procurement of the heart allograft with immediate placement on an ex vivo machine perfusion pump. This requires the collection of approximately 1.5 L of donor whole blood for priming of the pump circuit. Donor blood is collected passively via a large cannula placed in the right atrium, during which time the abdominal team must withhold initiation of cold perfusion. Although the collection of donor blood typically results in a short delay of 90 to 120 s, the process can add up to 5 min and has anecdotally resulted in organ loss because of extended delay.^1,5^

Further technical considerations require coordination between thoracic and abdominal teams, including patient positioning if a steep Trendelenburg position is required to aid in passive blood collection, utilization of suction-assisted collection of donor blood,^6^ definitions of functional warm ischemia time and time limits, and locations of an aortic cross-clamp and venous venting.^5,7^

In contrast, thoracoabdominal NRP further modifies prior abdominal NRP protocols by rapidly restoring and maintaining thoracoabdominal organ perfusion using an extracorporeal membrane oxygenated circuit via central aortic and right atrial cannulation.^8^ The abdominal team will either remain on standby or place abdominal cannulas as a backup in case of failure to initiate NRP. The great vessels of the aortic arch are clamped to eliminate cerebral reperfusion, although some authors argue that the great vessels should be ligated and divided with distal ends left open to confirm the absence of collateral cerebral perfusion.^9^ After reestablishment of in situ organ perfusion, the typical operative urgency of DCD is obviated, allowing for a controlled laparotomy and procurement similar to donation after brain death (DBD) donors. Both thoracic and abdominal organs are monitored by visual assessment and serial laboratory tests for 1 to 4 h before weaning from the extracorporeal circuit support to assess for cardiac function. As the circulatory support is discontinued, organs are perfused in situ by the heart before coordinating the timing of the aortic cross-clamp and immediate cold perfusion of preservative solution.^2,10^ The limited period of agonal ischemia with rapid restoration of perfusion via this approach may mitigate the severity of ischemia–reperfusion injury, allowing organs to recover while in situ during the donor operation.^11-14^ Although the use of NRP has been widely accepted in Europe, some debate persists regarding NRP within US practice regarding the ethical implications of in situ perfusion and definitions of the “dead donor” rule.^15-18^

Importantly, the effects of these modifications to existing DCD procurement techniques on graft and patient outcomes in the United States are not well described. Despite an early report describing unchanged graft utilization after combined TA-DCD procurement, the full impact of TA-DCD techniques on liver allograft outcomes and organ utilization is not completely understood.^19^ This study uses the national United Network for Organ Sharing (UNOS)/Organ Procurement and Transplantation Network (OPTN) database data to identify TA-DCD donors and evaluate liver utilization and posttransplant outcomes for patients who received a liver transplant after TA-DCD procurement.

## MATERIALS AND METHODS

All cardiac DCD transplantations performed between December 2019 and June 2021 were identified using a search of the UNOS/OPTN Standard Transplant Analysis and Research databases, using the most recent release as of September 2022. Study enrollment dates were capped to allow for at least 1 y follow-up from the time of transplant for all recipients. Database records were used to identify donor demographics, procurement data, disposition data for recovered livers, recipient demographics, and liver transplant recipient outcomes. All cardiac DCD donors were included in the TA-DCD study group if a liver was procured with intent for transplant at the time of recovery. Control groups were established using abdominal-only, noncardiac DCD (A-DCD) and DBD livers procured with intent for transplant during the study interval. Livers procured for pediatric, split-liver, and retransplant recipients were excluded from this study. Recorded time points in the UNOS/OPTN database were used to calculate the total ischemic time (withdrawal of life-sustaining treatment to initiation of preservation), functional warm ischemia time (onset of organ hypoperfusion to initiation of preservation), and asystolic ischemic time (declaration of asystole to initiation of preservation). The agonal phase was defined per UNOS criteria, with the onset at donor systolic blood pressure of <80 mm Hg or oxygen saturation of <80%. Because the use of DPP or NRP for DCD heart procurement is not included as a variable in the database, TA-DCD donors were stratified as DPP or NRP based on the duration of functional warm ischemia time. The investigative group selected a cutoff of ≤45 min based on procurement experience, organ acceptance criteria, and current NRP protocols.

The primary outcomes were posttransplant patient and graft survival. Secondary outcomes included utilization rate of livers procured with intent for transplant, indications for organ discard, including discard for warm ischemia time, and rates of organ discard stratified by technique of procurement. Liver utilization rates were defined as the number of livers successfully transplanted within all livers recovered with intent for transplant. Liver disposition and reasons for discard were obtained from the UNOS/OPTN database.

Statistical analysis was performed using Kruskal-Wallis tests between donor groups for continuous variables, as appropriate, and chi-square tests for categorical variables. Donor and recipient data were described as the median and interquartile range (IQR). Kaplan-Meier methods with log-rank testing were used to evaluate patient and graft survival. A *P* value of <0.05 was considered significant. Data analysis was performed using STATA (StataCorp LLC, College Station, TX) and JMP (JMP Statistical Discovery LLC, Cary, NC).

The Institutional Review Board at Cedars-Sinai Medical Center approved this study.

## RESULTS

### Donor Identification and Demographics

During the study period, 223 cardiac DCD transplants were identified. Fifty-five of these cardiac DCD donors were excluded from consideration for liver transplantation based on preoperative donor history and workup, with an additional 8 donors (7 DPP, 1 NRP) being declined intraoperatively without liver recovery. Ultimately, 160 donors proceeded to simultaneous TA-DCD procurement of heart and liver allografts. For TA-DCD donors, 135 procurements were completed with DPP and 25 were completed with NRP. During the study interval, 1706 A-DCD and 12 840 DBD donor livers were likewise procured with intent for transplant. Donor demographics and procurement data are summarized in Table 1.

**TABLE 1.** Donor demographics by donor type

Category	TA-DCD	A-DCD	DBD	*P*	
No. of donors	160	1706	12 840	Overall	TA-DCD vs A-DCD
Demographics					
Age, y, median (IQR)	28 (22.5–34)	40 (30–50)	41 (28–55)	<0.001	<0.001
Gender, n (%)				<0.001	<0.001
Male	140 (87.5%)	1120 (65.7%)	7710 (60.0%)		
Female	20 (12.5%)	586 (34.3%)	5130 (40.0%)		
Race/ethnicity, n (%)				<0.001	0.305
White, non-Hispanic	126 (78.8%)	1291 (75.7%)	7921 (61.7%)		
Black, non-Hispanic	20 (12.5%)	160 (9.4%)	2368 (18.4%)		
Hispanic/Latino	10 (6.3%)	197 (11.6%)	2049 (16.0%)		
Asian, non-Hispanic	2 (1.3%)	40 (2.3%)	348 (2.7%)		
Native American	1 (0.6%)	12 (0.7%)	75 (0.6%)		
Hawaiian/Pacific Islander	0 (0%)	2 (0.1%)	36 (0.3%)		
Multiracial	1 (0.6%)	4 (0.2%)	43 (0.3%)		
BMI, median (IQR)	26.01 (23.70–29.92)	27.17 (23.40–31.74)	27.26 (23.44–32.04)	0.198	0.095
Mechanism, n (%)				<0.001	<0.001
Anoxia	71 (44.4%)	935 (54.8%)	5854 (45.6%)		
CVA/stroke	7 (4.4%)	318 (18.6%)	3449 (26.9%)		
Head trauma	77 (48.1%)	391 (22.9%)	3251 (25.3%)		
CNS tumor	0 (0%)	0 (0%)	37 (0.3%)		
Other	5 (3.1%)	62 (3.6%)	249 (1.9%)		
Increased risk for blood-borne disease transmission, n (%)	37 (23.1%)	469 (27.5%)	3410 (26.6%)	0.430	0.235
Donor terminal laboratory values, median (IQR)					
Bilirubin, mg/dL	0.6 (0.4–0.9)	0.5 (0.4–0.8)	0.6 (0.4–1.0)	<0.001	0.0027
AST, U/L	49 (32.5–75.5)	52 (31–83)	41 (24–85)	<0.001	0.666
ALT, U/L	39 (25–76.5)	40 (22–75)	39 (21–79.5)	0.797	0.563
Creatinine, mg/dL	0.79 (0.62–1.01)	0.8 (0.6–1.2)	1.13 (0.79–2.02)	<0.001	0.767
pH	7.43 (7.40–7.46)	7.42 (7.37–7.46)	7.41 (7.37–7.45)	<0.001	0.035
Steatosis, median (IQR)					
No. recorded	25	88	436		
Microsteatosis	0% (0%–5%)	0% (0%–10%)	5% (0%–10%)	<0.001	0.831
Macrosteatosis	5% (0%–10%)	5% (0%–15%)	5% (0%–15%)	0.607	0.366

A-DCD, abdominal-only donation after circulatory determination of death; ALT, alanine aminotransferase; AST, aspartate aminotransferase; BMI, body mass index; CNS, central nervous system; CVA, cerebral vascular accident; DBD, donation after brain death; IQR, interquartile range; TA-DCD, thoracoabdominal donation after circulatory determination of death.

TA-DCD donors were younger (median 28 y; IQR, 22.5–34) than A-DCD (40 y; IQR, 30–50) and DBD (41 y; IQR, 28–55) donors at the time of donation (*P* < 0.001). A higher percentage of TA-DCD donors were male (87.5%) and Caucasian (78.8%) than A-DCD (65.7% and 75.7%, respectively) and DBD (60.0% and 61.7%, respectively) donors (*P* < 0.001 for both). Donors had similarly calculated body mass index across TA-DCD (26.01 kg/m^2^; IQR, 23.70–29.92), A-DCD (27.17 kg/m^2^; IQR, 23.40–31.74), and DBD (27.26 kg/m^2^; IQR, 23.44–32.04; *P* = 0.198) groups. When directly comparing TA-DCD to A-DCD donors, these demographic differences remained significant, except for similar race/ethnicity profiles between the 2 donor groups (*P* = 0.305; Table 1).

Terminal laboratory values recorded before organ donation revealed similar alanine aminotransferase levels between groups: TA-DCD 39 U/L (IQR, 25–76.5), A-DCD 40 U/L (IQR, 22–75), and DBD 39 U/L (IQR, 21–79; *P* = 0.797). A-DCD donors had lower total bilirubin levels and higher aspartate aminotransferase levels (0.5 mg/dL [IQR, 0.4–0.8] and 52 U/L [IQR, 31–83], respectively) than TA-DCD (0.6 mg/dL [IQR, 0.4–0.9] and 49 U/L [IQR, 32.5–75.5]) or DBD (0.6 mg/dL [IQR, 0.4–1.0] and 41 U/L [IQR, 24–85]) donors (*P* < 0.001 for both). TA-DCD donors had lower creatinine (0.79 mg/dL; IQR, 0.62–1.01) than A-DCD (0.80 mg/dL; IQR, 0.60–1.20) or DBD (1.13 mg/dL; IQR, 0.79–2.02) at time of donation. A pairwise comparison of TA-DCD and A-DCD donors revealed similar aspartate aminotransferase (*P* = 0.666), alanine aminotransferase (*P* = 0.563), and creatinine (*P* = 0.767) levels at the time of procurement.

A liver biopsy was performed on 25 TA-DCD livers, 436 A-DCD livers, and 5640 DBD livers. For donors who underwent liver biopsy, DBD donor livers had a higher degree of microsteatosis (5%; IQR, 0%–10%) than TA-DCD (0%; IQR, 0%–5%) or A-DCD (0%; IQR, 0%–10%) livers (*P* < 0.001), with similar degrees of macrosteatosis across all 3 donor groups (*P* = 0.607). TA-DCD and A-DCD donors had similar levels of microsteatosis (*P* = 0.831) and macrosteatosis (*P* = 0.366) on a biopsy.

Procurement times were recorded for both TA-DCD and A-DCD groups and were stratified by type of TA-DCD procurement (DPP versus NRP) to account for the differences in procurement techniques (Table 2). NRP donors had longer recorded procurement intervals including total ischemia time (110 min; IQR, 76–150; *P* < 0.001), functional warm ischemia time (102 min; IQR, 77–142 min; *P* < 0.001), and asystolic ischemia time (80 min; IQR, 61–124 min; *P* < 0.001); however, this was expected because of the inclusion of regional perfusion time within the definition of traditional DCD time intervals. When comparing A-DCD with DPP donors, total ischemia time (23 min [IQR, 19–28 min] versus 24 min [IQR, 21–27 min]; *P* = 0.343), functional ischemia time (20 min [IQR, 16–24 min] versus 21 min [IQR, 18–24 min]; *P* = 0.277), and asystolic ischemia time (6 min [IQR, 4–8 min] versus 6 min [IQR, 4–8 min]; *P* = 0.05) were found to be similar.

**TABLE 2.** Procurement times, organ utilization rates, and indications for discard by donor type (above) and detailed description of 27 discarded TA-DCD livers (below)

	TA-DCD			A-DCD (n = 1706)	DBD (n = 12 840)	*P*	
	Overall (n = 160)	DPP (n = 135)	NRP (n = 25)				
Procurement times, median (IQR)						Overall	DPP vs A-DCD
Total ischemic time, min		24 (21–27)	110 (76–150)	23 (19–28)		<0.001	0.343
Functional warm ischemia time, min		21 (18–24)	102 (77–142)	20 (16–24)		<0.001	0.277
Asystolic ischemia time, min		6 (4–8)	80 (61–124)	6 (4–8)		<0.001	0.05
Organ utilization						*P*	
Organ utilization rate	133/160 (83.1%)	109/135 (80.7%)	24/25 (96%)	12 19/1706 (71.4%)	11 952/12 840 (93.0%)	<0.001	
No. of discarded organs	27	26	1	487	888		
Organ discard rate	16.9%	19.3%	4%	28.5%	6.9%		
Indications for discard						<0.001	
Warm ischemia time	10 (37.0%)	9 (34.6%)	1 (100%)	107 (22.0%)	5 (0.6%)		
Too old on ice	0 (0%)	0 (0%)	0 (0%)	4 (0.8%)	19 (2.1%)		
Vascular damage	1 (3.7%)	1 (3.8%)	0 (0%)	3 (0.6%)	15 (1.7%)		
Donor medical HTx	0 (0%)	0 (0%)	0 (0%)	0 (0%)	5 (0.6%)		
Hepatitis	0 (0%)	0 (0%)	0 (0%)	3 (0.6%)	1 (0.1%)		
Organ trauma	0 (0%)	0 (0%)	0 (0%)	3 (0.6%)	7 (0.8%)		
Organ not as described	0 (0%)	0 (0%)	0 (0%)	0 (0%)	5 (0.6%)		
Biopsy result	1 (3.7%)	1 (3.8%)	0 (0%)	78 (16.0%)	364 (41.0%)		
Unsuitable for Tx in OR	0 (0%)	0 (0%)	0 (0%)	4 (0.8%)	39 (4.4%)		
Poor organ function	1 (3.7%)	1 (3.8%)	0 (0%)	19 (3.9%)	28 (3.2%)		
Infection	0 (0%)	0 (0%)	0 (0%)	2 (0.4%)	0 (0%)		
Diseased organ	2 (7.4%)	2 (7.7%)	0 (0%)	26 (5.3%)	53 (6.0%)		
Anatomical abnormalities	1 (3.7%)	1 (3.8%)	0 (0%)	24 (4.9%)	75 (8.4%)		
List exhausted	2 (7.4%)	2 (7.7%)	0 (0%)	38 (7.8%)	55 (6.2%)		
Other	9 (33.3%)	9 (34.6%)	0 (0%)	176 (36.1%)	217 (24.4%)		
**Type of TA-DCD**	**Reason for discard**		**Total ischemic time, min**	**Functional warm ischemia time, min**	**Asystolic ischemia time, min**		
DPP	Warm ischemia time		41.00	37.00	NR		
DPP	Warm ischemia time		23.00	21.00	3.00		
DPP	Warm ischemia time		27.00	26.00	8.00		
DPP	Warm ischemia time		36.00	31.00	4.00		
DPP	Warm ischemia time		27.00	25.00	5.00		
DPP	Warm ischemia time		75.00	35.00	8.00		
DPP	Warm ischemia time		32.00	22.00	10.00		
DPP	Warm ischemia time		22.00	19.00	2.00		
DPP	Warm ischemia time		31.00	27.00	4.00		
DPP	Liver laceration		36.00	26.00	5.00		
DPP	Liver laceration		22.00	20.00	8.00		
DPP	Surgical injury		23.00	23.00	9.00		
DPP	Vascular injury		27.00	26.00	11.00		
DPP	Biopsy Results		17.00	16.00	5.00		
DPP	Diseased organ		24.00	21.00	10.00		
DPP	Diseased organ		21.00	15.00	NR		
DPP	Poor organ function		27.00	22.00	3.00		
DPP	Ruled out after evaluation in OR		21.00	20.00	3.00		
DPP	Did not perform well on pump		24.00	22.00	7.00		
DPP	Poor flush on backtable		19.00	13.00	7.00		
DPP	Poor flush		26.00	24.00	9.00		
DPP	Liver did not flush well		30.00	29.00	5.00		
DPP	Poor backtable flush		31.00	28.00	3.00		
DPP	Anatomical abnormalities		21.00	18.00	7.00		
DPP	List exhausted		15.00	14.00	3.00		
DPP	List exhausted		24.00	20.00	5.00		
NRP	Warm ischemia time		102.00	102.00	78.00		

A-DCD, abdominal-only donation after circulatory determination of death; DBD, donation after brain death; DPP, direct procurement and perfusion; HTx, heart transplantation; IQR, interquartile range; NR, not reported; NRP, normothermic regional perfusion; OR, operation room; TA-DCD, thoracoabdominal DCD; Tx, transplantation.

### Liver Utilization Rates

One hundred thirty-three of 160 TA-DCD livers proceeded to transplant for an overall organ discard rate of 16.9%. DBD donors had a lower organ discard rate (888/12 840; 6.9%) than A-DCD (487/1706; 28.5%) or TA-DCD donors (*P* < 0.001). When comparing DCD procurement types, NRP donors had a lower rate of organ discard (1/25; 4%) than DPP (25/135; 19.3%) or A-DCD (1219/1706; 28.5%) donors (*P* = 0.002). Indications for organ discard are summarized in Table 2. TA-DCD livers had a significantly higher rate of organ discard for warm ischemia time (10/27 livers; 37.0%) than A-DCD (107/487 livers; 22.0%) or DBD (5/888 livers; 0.6%; *P* < 0.001). A summary of all indications for organ discard among rejected TA-DCD livers is included in Table 2. In addition to 10 organ discards for warm ischemia time, an additional 4 livers (14.8%) were discarded for surgical injury or liver laceration within the DPP procurement group.

### Recipient Demographics and Outcomes

Overall, DBD liver recipients were younger (47 y old, IQR, 48–64) than TA-DCD (59 y old, IQR, 52–65) and A-DCD (59 y old; IQR, 52–65) recipients (*P* < 0.001). TA-DCD recipients were more frequently male (70.7%) and Caucasian (82.3%) than A-DCD (66.9% and 74.2%, respectively) and DBD (63.5% and 69.9%, respectively) recipients (*P* < 0.001 for both). Recipients across all groups had similarly calculated body mass index (*P* = 0.085). The primary indication for transplant was alcoholic liver disease for all study groups, and more patients were transplanted for hepatocellular carcinoma in the DBD recipient group (43.8%) than in the TA-DCD (25.6%) or A-DCD (21.7%) groups (*P* < 0.001). DBD liver recipients had higher model for end-stage liver disease scores at the time of transplant (25.2; IQR, 16.9–31.6) than TA-DCD (19.1; IQR, 12.4–25.3) or A-DCD (18.6; IQR, 13.2–23.5) recipients (*P* < 0.001). DBD liver recipients also had higher rates of ascites (44.5%) than TA-DCD (32.3%) or A-DCD (30.8%) recipients (*P* < 0.001). TA-DCD livers had a shorter cold ischemia time (median 5.1 h; IQR, 4.5–6.4) than A-DCD (5.3 h; IQR, 4.4–6.3) or DBD (5.8 h; IQR, 4.8–7.0; *P* < 0.001) livers. Recipient data are summarized in Table 3.

**TABLE 3.** Recipient demographics and organ outcomes by donor type

Category	TA-DCD	A-DCD	DBD	*P*
No. of recipients	133	1219	11952	
Demographics				
Age, y, median (IQR)	59 (52–65)	59 (52–65)	47 (48–64)	<0.001
Gender, n (%)				
Male	94 (70.7%)	815 (66.9%)	7586 (63.5%)	0.012
Female	39 (29.3%)	404 (33.1%)	4366 (36.5%)	
Race/ethnicity, n (%)				<0.001
White, non-Hispanic	107 (82.3%)	905 (74.2%)	8372 (69.9%)	
Black, non-Hispanic	3 (2.3%)	65 (5.3%)	854 (7.1%)	
Hispanic/Latino	18 (13.8%)	187 (15.3%)	2006 (16.8%)	
Asian, non-Hispanic	0 (0%)	37 (3.1%)	504 (4.2%)	
Native American	0 (0%)	12 (1.0%)	117 (1.0%)	
Hawaiian/Pacific Islander	1 (0.8%)	2 (0.2%)	19 (0.2%)	
Multiracial	1 (0.8%)	11 (0.9%)	80 (11.0%)	
BMI, median (IQR)	28.7 (24.4–32.6)	28.8 (25.1–33.0)	28.3 (24.5–32.7)	0.0852
Etiology				<0.0001
HCV	27 (20.8%)	188 (15.4%)	1340 (11.2%)	
EtOH	45 (34.6%)	396 (32.5%)	4663 (39.0%)	
NAFLD	28 (21.5%)	350 (28.7%)	2525 (21.1%)	
HBV	4 (3.1%)	27 (2.2%)	274 (2.3%)	
Cholestatic	10 (7.7%)	114 (9.4%)	1196 (10.0%)	
Other	16 (12.3%)	144 (11.9%)	1954 (16.4%)	
Recipient complexity				
MELD at transplant	19.1 (12.4–25.3)	18.6 (13.2–23.5)	25.2 (16.9–31.6)	<0.001
HCC diagnosis	34 (25.6%)	265 (21.7%)	5647 (43.8%)	<0.001
Ascites	32.3%	30.8%	44.5%	<0.001
PV thrombus	13.0%	12.7%	13.2%	0.538
Multivisceral transplant				
Liver-kidney	13.0%	10.0%	11.0%	0.397
Heart-liver	0%	0%	0.7%	0.007
Liver cold ischemia time	5.1 (4.5–6.4)	5.3 (4.4–6.3)	5.8 (4.8–7.0)	<0.001
Outcomes				
Patient survival				0.893
6 mo	94.7%	95.0%	95.0%	
12 mo	92.3%	92.6%	93.0%	
24 mo	90.4%	86.6%	87.0%	
Graft survival				0.009
6 mo	93.2%	91.7%	93.6%	
12 mo	90.1%	88.8%	91.5%	
24 mo	85.1%	82.1%	85.3%	
Length of stay, d, median (IQR)	8 (6–13)	8 (6–13)	10 (7–17)	<0.001

A-DCD, abdominal-only donation after circulatory determination of death; BMI, body mass index; DBD, donation after brain death; EtOH, ethanol; HBV, hepatitis B virus; HCC, hepatocellular carcinoma; HCV, hepatitis C virus; IQR, interquartile range; MELD, model for end-stage liver disease; NAFLD, nonalcoholic fatty liver disease; PV, portal vein; TA-DCD, thoracoabdominal DCD.

**FIGURE 1.**
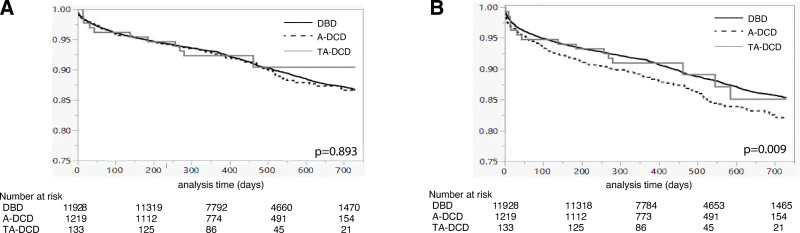
Kaplan-Meier analysis demonstrating equivalent patient survival for (A) patients receiving DBD, A-DCD, and TA-DCD liver allografts (*P* = 0.893). B, Patients receiving liver allografts from TA-DCD and DBD donors showed superior graft survival compared with A-DCD liver transplants (*P* = 0.009). A-DCD, abdominal-only donation after circulatory determination of death; DBD, donation after brain death; TA-DCD, thoracoabdominal DCD.

The median duration of follow-up for liver recipients was 377 d (IQR, 358–705) in the TA-DCD group, 378 d (IQR, 356–713) in the A-DCD group, and 378 d (IQR, 356–707) in the DBD group. Overall patient survival rates at 12 and 24 mo were similar between TA-DCD (92.3% and 90.4%), A-DCD (92.6% and 86.6%), and DBD (93.0% and 87.0%) liver recipients during the follow-up interval (*P* = 0.893; Figure 1A). Graft survival rates at 12- and 24 mo were lower after A-DCD liver transplants (88.8% and 82.1%) than TA-DCD (90.1% and 85.1%) and DBD (91.5% and 85.3%) transplants (*P* = 0.009; Figure 1B). Hospital length of stay was shorter for the TA-DCD (8 d; IQR, 6–13) and A-DCD (8 d; IQR, 6–13) than for the DBD group (10 d; IQR, 7–17; *P* < 0.001).

## DISCUSSION

In this study, we used the national UNOS/OPTN database to retrospectively study whether the techniques used for cardiac DCD procurement affected liver transplant outcomes or liver allograft utilization. Specifically, this study focused on livers that were procured with the intent of transplant. By eliminating analysis of donors deemed unacceptable for liver donation before procurement event or declined intraoperatively without recovery, this study design aims to isolate the effect of procurement techniques on organ utilization, independent of underlying donor quality. Including both A-DCD and DBD donor control groups allows for a complete understanding of organ utilization and outcomes during the study period.

Overall, patient survival in the TA-DCD group was similar to outcomes for A-DCD and DBD liver transplants at 1 and 2 y posttransplant. This study also demonstrates that patients undergoing A-DCD liver transplants have inferior graft outcomes to TA-DCD and DBD at 1 and 2 y posttransplant. This primary outcome suggests that excellent liver transplant outcomes are maintained when TA-DCD is well executed by either DPP or NRP. This study reports the longest-term outcomes for these liver recipients, with sufficient follow-up to capture delayed indications for retransplantation, including ischemic cholangiopathy. The superior graft outcomes seen in TA-DCD are likely representative of the highly selected population of donors, with superior organ quality and freedom from injury to donate both heart and liver allografts. Although not studied within the context of this article, only 160 of the 223 cardiac DCD donors (72%) during the study period were considered for liver donation at the time of procurement. Given the outstanding graft and patient outcomes from TA-DCD donors, further investigation is warranted to identify and eliminate barriers to liver donation from this highly selected population of donors.

The overall impact of TA-DCD procurement techniques on liver transplants must also consider organ utilization and indications for organ discard. The previous study by Feizpour et al^19^ identified an overall increase in observed liver utilization compared with what was expected, providing important information to the transplant community that TA-DCD donation was not resulting in the increased liver discard. However, the present study has revealed that although the overall rate of organ discard is similar between TA-DCD and A-DCD donors, the indications for liver discard were significantly different between donor groups. TA-DCD donors had a higher rate of discard for prolonged warm ischemia (37%) than A-DCD (22%) or DBD (0.6%). This was particularly evident with the DPP technique, because 9 of 10 organ discards within the TA-DCD group for prolonged warm ischemia time occurred during DPP procurements, whereas only 1 organ procured via NRP was discarded for prolonged warm ischemia time.

This increase in discard for an indication of warm ischemia time is difficult to reconcile when considering that A-DCD and DPP donors had nearly identical procurement time intervals, particularly during functional and asystolic warm ischemia time phases. Because this is the first series of TA-DCD procurements, this trend toward organ discard may reflect a learning curve and developing levels of comfort with the TA-DCD procurement techniques. Recipient surgeons may have been less likely to accept a liver for transplant if they perceived a longer functional warm ischemia time as a result of simultaneous cardiac DCD procurement. Liver utilization from TA-DCD donors should be studied prospectively in the current era because thoracoabdominal procurements have become routine across the country.

When fully considering the effects of procurement type on organ utilization, it should also be recognized that an additional 15% of livers procured by DPP were discarded for liver laceration or vascular injury during procurement. Even if DPP does not add significantly to organ discard for warm ischemia time, the additional loss of organs because of procurement injury suggests that further modifications to DPP protocols may be needed to improve organ utilization. Like A-DCD procurements, DPP procurements carry a level of urgency to minimize warm ischemia time, with the added complexity of 2 procurement teams working in close proximity with differing objectives. Rates of liver injury during DPP procurements should be further investigated, with coordination between thoracic and abdominal teams to protect the liver from iatrogenic injury and discard.

The early results from NRP procurements performed in this study present a promising path forward to successfully optimize TA-DCD procurements. NRP donors had the lowest rate of organ discard (1/25; 4%) for all DCD procurement groups, with the only discard attributable to warm ischemia time and no organ injury leading to discard. Procurement data specific to NRP procurements, particularly the time to proceed from declaration of death to initiation of extracorporeal circuit support, are not currently available in UNOS/OPTN database records. However, the low rate of organ discard suggests that the combination of rapid cannulation by a single team, coupled with the opportunity to observe the organ for effects of ischemia–reperfusion injury, may increase surgeon comfort in using DCD organs from NRP donors with extended functional warm ischemia time.

Because machine perfusion has revolutionized cardiac DCD transplantation, the broader use of machine perfusion in DCD liver transplant may also impact the utilization of TA-DCD livers. Because both hypothermic and normothermic machine perfusion have been shown to decrease rates of ischemic cholangiopathy after transplant, the addition of liver machine perfusion to TA-DCD transplants may reduce marginal organ discard for prolonged warm ischemia time.^20,21^ The use of machine perfusion in liver transplantation should be monitored along with UNOS/OPTN repository data to fully characterize organ outcomes.

The findings in this study are limited by the small sample size in this early US experience (particularly in NRP), as well as the nature and shortcomings of repository data. Continued expansion of TA-DCD procurements will elucidate the benefits of DPP versus NRP techniques. Other important features of graft outcomes, such as prevalence and severity of ischemic cholangiopathy, are not fully captured in the national UNOS/OPTN database and require further study. Importantly, given the rapid incorporation of TA-DCD procurements into clinical practice, UNOS/OPTN data collection will need to rapidly adapt to reflect and capture current donor practices. Additional fields should be added to UNOS/OPTN donor data collection, including identification of TA-DCD donors, type of TA-DCD procurement (DPP versus NRP) used, and procurement-specific time points, including incision time, time to collect donor blood in DPP procurements, and time to the initiation of extracorporeal circulation for NRP procurements. The designation of the TA-DCD procurement type is of particular importance because this will be critical to investigating these long-term outcomes. Although our designation of DPP or NRP donor was set by the authors based on a time cutoff, the distinct disparity in agonal-to-cross clamp times seen between the 2 groups supports the accuracy of this designation. Furthermore, single-center reports of NRP experience correlate with the donor centers listed in the UNOS/OPTN database—again supporting the accuracy of these designations.^4,22^

This study is among the first reports of TA-DCD outcomes in the United States and is the first to report a direct comparison with abdominal-only procurements. The results of this study support excellent liver transplant survival outcomes for allografts from TA-DCD donors and provide important insights into TA-DCD procurement techniques. Our common goal in transplantation is to maximize organ access and optimize organ outcomes. Continued application of TA-DCD requires close collaboration between thoracic and abdominal transplant communities to maintain these outcomes in a unified manner.

## ACKNOWLEDGMENTS

The authors acknowledge and honor the memory of our colleague Vinay Sundaram, who contributed to this project and was pivotal in liver transplantation research at Cedars-Sinai.

## Supplementary Material


