# Supplementary figures and images for: Preserved 2-y Liver Transplant Outcomes Following Simultaneous Thoracoabdominal DCD Organ Procurement Despite Effects on Liver Utilization Rate
Source: Transplant Direct. 2023 Oct 20;9(11):e1528. doi: 10.1097/TXD.0000000000001528 (PMC10593259; doi:10.1097/TXD.0000000000001528)

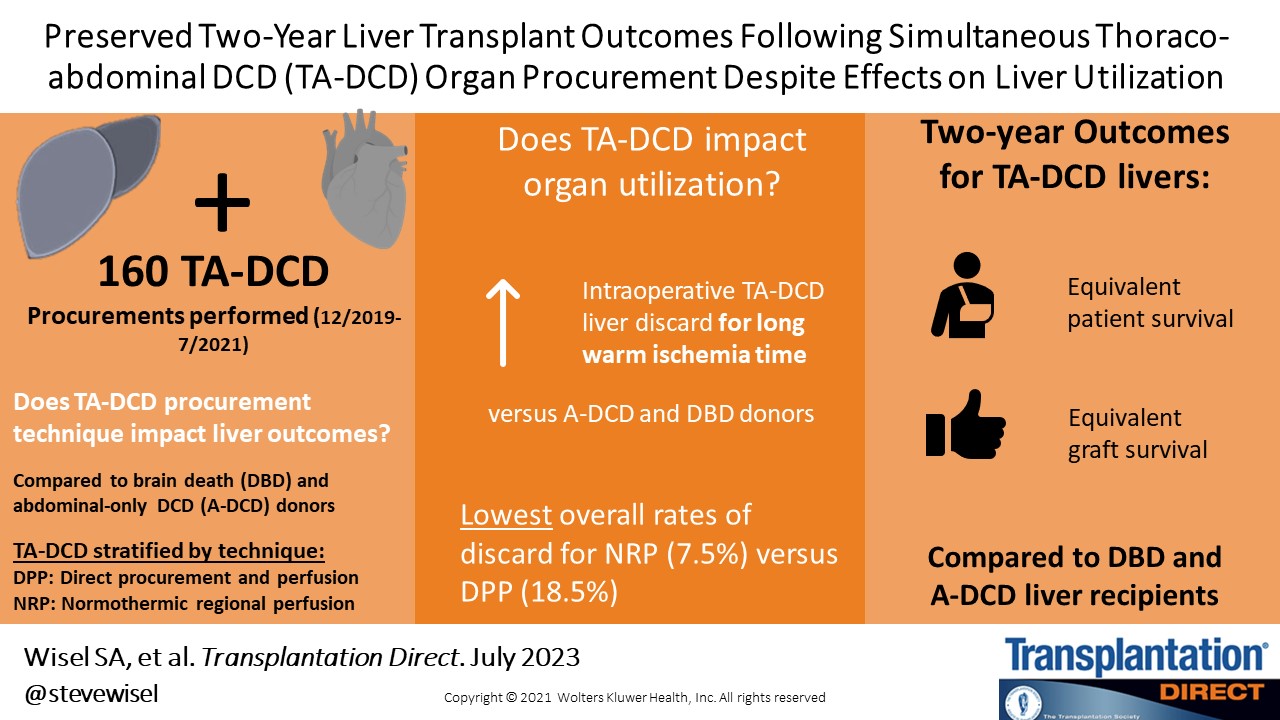

Supplement: Supplementary file 1 [file txd-9-e1528-s001.jpg]
